# BrowZine

**DOI:** 10.5195/jmla.2017.259

**Published:** 2017-10-01

**Authors:** Elizabeth G. Hinton

## PURPOSE

Established in 2011, BrowZine is a “Journal Enhancement Platform” owned by Third Iron. Usable from a desktop, laptop, or mobile device, the journal browsing service allows users to create a custom electronic journal library of their favorite journals. BrowZine does not change the journal reading experience but helps users discover and organize their favorite journals in a library’s existing electronic journal collection.

## DESCRIPTION AND USES

With a variety of available subjects and subcategories (e.g., arts and humanities, history, law, mathematics, biomedical and health sciences), BrowZine is not limited to health sciences libraries. Brow-Zine’s predictive search can be used multiple ways: searching by subject, title, or ISSN. In subject browsing mode, the user can either view all available journals in a specific subject area or further limit to a specific discipline within that subject area.

**Figure 1:**
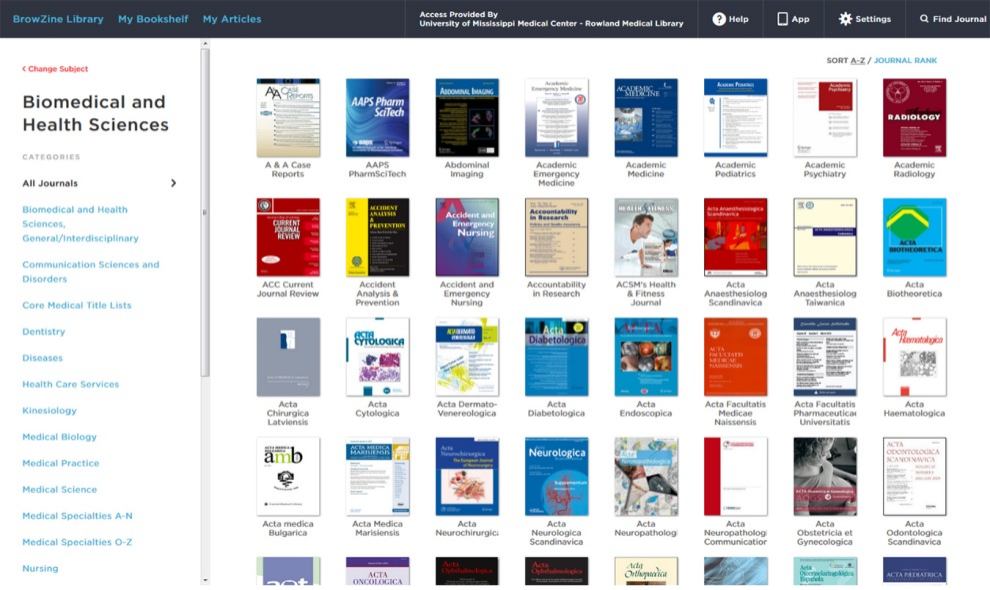
BrowZine Biomedical and Health Sciences subcategories

As an example, through the reviewer’s institutional access, “Biomedical and Heath Sciences” can be limited to dentistry, health care services, nutrition, veterinary science, or any of the additional seventeen subcategories (Figure 1). From there, journal titles can be displayed in alphabetical order or by SCImago journal rank. Brow-Zine supports hundreds of publishers, which, by Third Iron’s best estimate, equates to tens of thousands of individual journal titles [1].

BrowZine uses a custom 3-tier taxonomy [2] of more than 1,200 terms based on several existing taxonomies: the National Academies’ Taxonomy of Fields, the National Center for Education Statistics Classification of Instructional Programs, the Berkeley Press 2011 Taxonomy of Academic Programs, Library of Congress subject headings, and Medical Subject Headings (MeSH). BrowZine also keeps “bookcases” of core journal lists: the Essential Core and Research Level Core lists maintained by the Public Heath/Health Administration Section of the Medical Library Association (MLA) [3], the Abridged Index Medicus (AIM) core clinical journals classified in MEDLINE/PubMed [4], and the MLA Veterinary Medical Libraries Section’s Basic List of Veterinary Medical Serials [5].

## INTENDED AUDIENCE

Appropriate for students, physicians, faculty, and librarians, BrowZine is useful for anyone who wants to browse journals on an intuitive platform. BrowZine markets itself to academic libraries, medical libraries, and corporate information centers, so the user possibilities are seemingly endless. According to the Third Iron website, BrowZine is used at nearly 500 libraries and information centers in 16 countries [6]. In the medical field, Third Iron promotes Brow-Zine as a service for busy clinicians to keep up with the published literature. In fact, at the reviewer’s own institution, one of BrowZine’s biggest champions is a clinical faculty member who recommends the product to residents and fellows as a way to stay current.

Finding a champion in the institution is important, because BrowZine can be relatively challenging to market. It is difficult to adequately describe the product to potential users on a library’s website, which makes product demonstration crucial. But because many of BrowZine’s intended users are busy clinicians, reaching members of this group to provide one-on-one demonstrations might be an impossible task. In addition to the strategy of recruiting several clinicians or faculty members to help promote the service to their students and colleagues, libraries can use marketing tools provided by Third Iron to promote the product: a LibGuide, widgets, images, and several printable posters.

## MAJOR FEATURES

BrowZine’s features can be tailored to suit users’ preference. In addition to simply browsing favorite journals, users can create reading lists; save articles; share articles through Twitter, Facebook, LinkedIn, or email; and export citations to Zotero, Mendeley, BibTex, or EndNote. Articles can also be saved in BrowZine under My Articles. Clicking on the article’s title will automatically connect users to an article’s full text using the library’s existing linking tool.

Perhaps BrowZine’s most notable feature is My Bookshelf. My Bookshelf consists of four colored rooms, or “bookcases,” that hold sixteen titles each (four on each “shelf”), for a total of sixty-four individual journals. The user can change titles of bookcases and shelves to aid in organization. Any journal can easily be added to My Bookshelf from the journal’s table of contents page by clicking “Add to My Bookshelf.” When the desktop application is open, BrowZine pushes out badge alerts for each journal title to indicate the number of unread articles.

Another useful feature of BrowZine is the ability to integrate the product into LibGuides. Examples include widgets directing users to predetermined subject collections, an A–Z search box, and tables of top journals with access to each title’s current issue. Third Iron has created its own LibGuide with resources and instructions for successfully adding BrowZine to a library’s existing LibGuide. New for 2017 is BrowZine’s application programming interface (API) functionality: the Journal Availability API and the Search Results API are currently available, and late 2017 will see release of the Article in Context API.

BrowZine’s mobile application (free with an institutional subscription) can be downloaded for Apple, Android, and Kindle Fire using each device’s respective store. Once a BrowZine username and password have been created, the app syncs easily with the desktop version. Similar to the functionality of the desktop version, the app can be set up to push alerts when new content is added to favorite journals.

## TECHNICAL REQUIREMENTS

BrowZine works with EZProxy, WAM proxy, OpenAthens, and virtual private network (VPN) authentication systems. A “Pairing Service” is also available for libraries without remote authentication. BrowZine can sync with most existing A–Z systems, including Serial Solutions 360, OCLC WorldCat, TDNet, Alma, and ProQuest Intota.

## USABILITY

Generally, new users should not require a great deal of training to use BrowZine. The service is easy to learn, and users should be able to create a BrowZine username and password and easily build collections on their own.

Although the product is intuitive, BrowZine offers several options for technical support, if the need arises. Support is available through email, or one may visit the Knowledge Base, an extensive bank of questions and answers covering general questions for users, information for publishers, video tutorials, and frequently asked questions about library configuration and working with library holdings. Additionally, BrowZine periodically hosts webinars to offer training and to highlight new product features, and a robust user community is always available to help further BrowZine’s evolution and improvement.

## RECOMMENDATION

User experience will vary, because the service depends on a user’s institutional holdings, and access to open access journals can be limited. However, BrowZine is a reasonably priced product with good overall value for libraries serving health sciences communities. A free trial of BrowZine with access to content from open access publishers is available through Third Iron’s website.
